# BeadWatch: Automated Longitudinal Quality Control Monitoring for Luminex Bead‐Based Immunoassays in Histocompatibility Laboratories

**DOI:** 10.1111/tan.70849

**Published:** 2026-07-10

**Authors:** Mian Chen, Hannah Docker, Deborah Williams, Gemma Cutland, Manuel Quirno Costa, Martin C. N. Barnardo

**Affiliations:** ^1^ Transplant Immunology Laboratory Churchill Hospital Oxford UK

**Keywords:** histocompatibility, Luminex, MFI, open‐source software, quality control, signal‐to‐noise ratio, single antigen beads

## Abstract

Luminex bead‐based immunoassays are central to HLA antibody testing in histocompatibility laboratories, yet QC monitoring remains largely manual. Although ISO 15189, EFI and ASHI standards require statistical trend analysis, laboratories typically rely on spreadsheet tracking or periodic visual inspection; these approaches can detect gross run failures but are poorly suited to surfacing gradual trends in instrument, reagent and operator performance. We developed BeadWatch, an open‐source automated QC dashboard application that reads directly from vendor databases, applies Levey‐Jennings charts with optional I‐MR views, and includes cross‐instrument and cross‐operator comparison dashboards and configurable alerting. BeadWatch currently supports OneLambda HLA Fusion and deploys as a single portable application with no installation steps. Single‐site deployment in our laboratory ingested approximately 20 years of historical data, revealing instrument lifecycle trajectories and a step change in instrument MFI following a platform transition—patterns consistent with known instrument behaviour but not previously documented. During prospective monitoring, an automated alert identified a pipetting error before the responsible operator had independently recognised the issue. BeadWatch is freely available at https://github.com/oxfordgenes/BeadWatch.

**Abbreviations**
API: application programming interface
ASHI: American Society for Histocompatibility and Immunogenetics
EFI: European Federation for Immunogenetics
H&I: histocompatibility and immunogenetics
I‐MR: individuals‐moving range
ISO: International Organisation for Standardisation
LJ: Levey‐Jennings
MFI: mean fluorescence intensity
NC: negative control
PC: positive control
QC: quality control
S/N: signal‐to‐noise ratio
SAB: single antigen bead
SD: standard deviation
SPC: statistical process control

## 1 Introduction

Luminex bead‐based solid‐phase immunoassays are the standard platform for HLA antibody testing in histocompatibility and immunogenetics (H&I) laboratories [1, 2]. The same Luminex xMAP platform also supports HLA typing via PCR sequence‐specific oligonucleotide probes (SSOP) [3], though BeadWatch targets the antibody testing workflow. These solid‐phase assays report mean fluorescence intensity (MFI) values whose clinical interpretation depends on analytical reproducibility [2], including in single‐antigen bead testing for HLA antibody specificities [4]. Drift in MFI baselines or signal‐to‐noise ratios can affect antibody assignment thresholds and the longitudinal comparability of results for sensitised patients.

### 1.1 Current QC Challenges

Despite the clinical importance of MFI reproducibility, QC practices for Luminex systems in H&I laboratories remain largely ad hoc. Accreditation standards such as ISO 15189:2022, EFI (v8.1) and ASHI (2025) [5, 6, 7], all require documented QC procedures designed to detect trends and shifts over time, yet the specific implementation is left to individual laboratories, and in practice most rely on:

- Manual spreadsheet tracking. QC values are recorded manually in spreadsheets after each run. This is labour‐intensive, error‐prone and provides no real‐time alerting.
- Vendor‐provided summary screens. Vendor software such as OneLambda HLA Fusion displays per‐run control values, but these are transactional, showing the current run without longitudinal trending or statistical overlays.
- Periodic lot‐to‐lot comparison. QC sample results may be compared across bead lots during qualification, but this is typically a one‐time assessment rather than continuous monitoring.

These approaches detect problems only when an operator actively reviews data, which may occur hours or days after the run. Several failure modes develop gradually and escape per‐run notice:

- Instrument drift. Laser degradation, optical ageing and fluidics wear produce slow MFI shifts that are invisible per‐run but clear in trend charts [8, 9].
- Inter‐lot variability. MFI differences between bead production lots can occur, sometimes affecting only a subset of beads. Even small offsets may shift effective antibody positivity cut‐offs for those beads. These differences appear normal in isolation but become evident when trended across lot transitions.
- Bead reactivity loss. Bead lots may lose reactivity over their shelf life due to storage conditions or antigen degradation, reducing positive signal strength.
- Elevated negative control. Reagent issues such as contaminated wash buffers, expired conjugates or preparation errors can raise negative control background, compressing the signal‐to‐noise ratio.
- Bead count decline. Probe blockage, fluidics issues or laser drift causing beads to fall outside acquisition gates can reduce bead counts. Below approximately 25 events per bead, MFI values become statistically unreliable [1].
- Operator variability. Despite standardised SOPs, minor differences in sample preparation technique can introduce systematic between‐operator variation that is invisible without comparative analysis

### 1.2 Statistical Process Control (SPC) in Clinical Laboratories

SPC, particularly Levey‐Jennings (LJ) charts and Westgard rules, is well established in clinical chemistry [10, 11]. However, adoption of formal SPC in H&I laboratories has been limited; vendor software does not provide longitudinal statistical monitoring, leaving most laboratories dependent on manual approaches that are difficult to scale, despite ISO 15189 explicitly recommending statistical techniques where practicable.

Matson et al. described shinyMBA, an R Shiny application applying LJ charts to Luminex assays in infectious disease serosurveillance [12]. While shinyMBA demonstrates SPC value for Luminex platforms, it targets infectious disease workflows using exported data files and does not address H&I‐specific needs such as reagent batch‐aware control selection, tracked‐bead monitoring or cross‐instrument comparison. To our knowledge, no published tool provides automated longitudinal QC monitoring tailored to histocompatibility Luminex workflows with direct vendor database integration.

### 1.3 Rationale for BeadWatch

We developed BeadWatch as a complementary monitoring layer that reads from vendor databases in read‐only mode. The current release includes a tested adaptor for OneLambda HLA Fusion (Thermo Fisher Scientific).

The starting point was to remove manual transcription: BeadWatch polls the vendor database at configurable intervals, then applies LJ trend visualisation, with mean and ±2SD reference lines across multiple configurable time windows, to several QC metrics in parallel, including control MFI, bead counts, signal‐to‐noise ratio and the performance of individually tracked beads. The same statistical treatment extends to between‐group variability through cross‐instrument and cross‐operator comparison dashboards, and to longitudinal monitoring of known‐reactivity QC samples. Two practical considerations shaped the rest of the design: BeadWatch is distributed as a single portable executable with read‐only vendor database access, so it can sit alongside any existing workflow without changing it, and is released open source to enable community adoption, audit and extension.

This paper describes BeadWatch's architecture, statistical methods and features and presents examples from single‐site deployment experience.

## 2 Material and Methods

### 2.1 System Architecture

**FIGURE 1:**
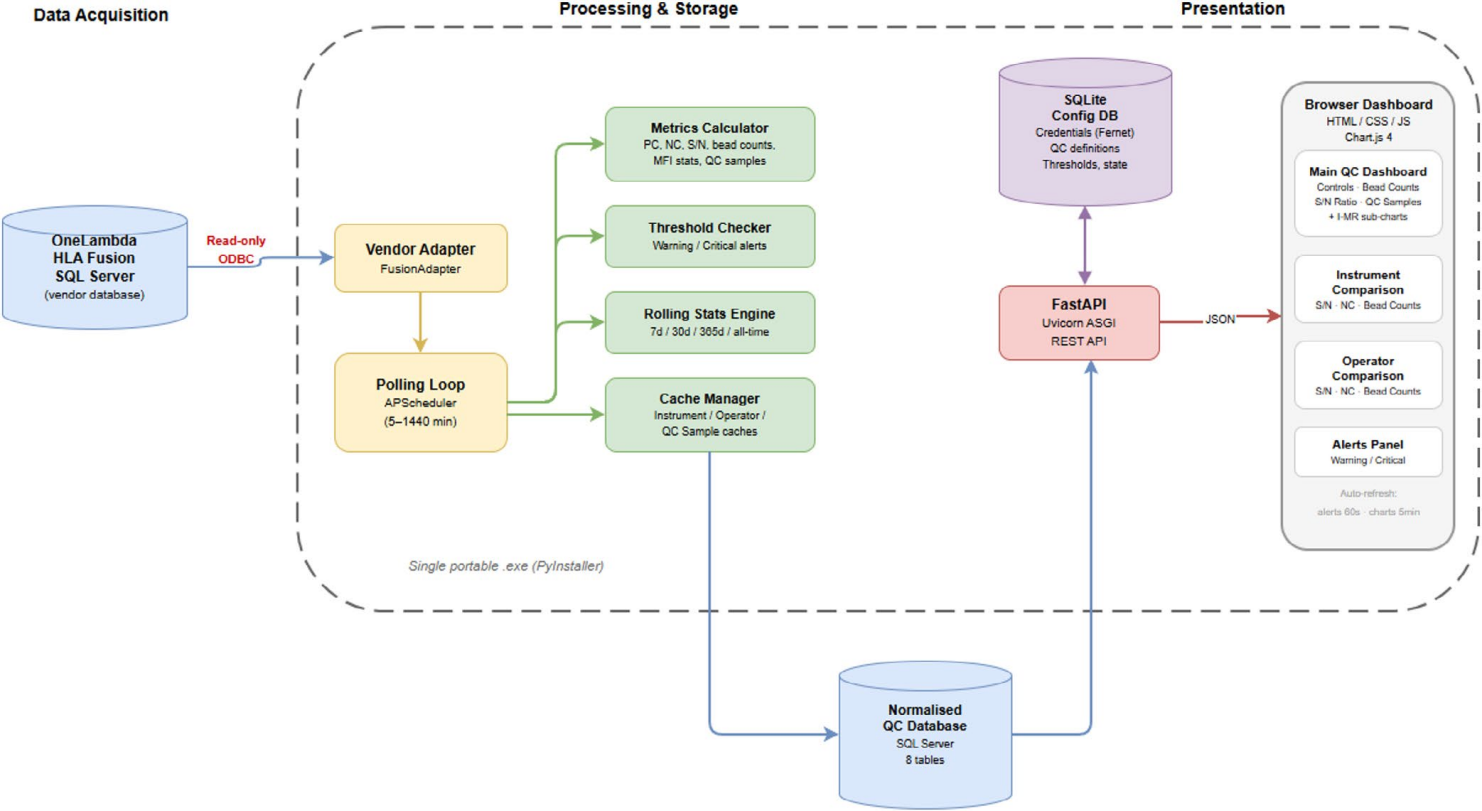
BeadWatch system architecture. The application spans three tiers: Data acquisition from the vendor SQL Server database via a read‐only ODBC connection, a processing tier for QC metric calculation and alert evaluation, and a browser‐based dashboard served via a FastAPI REST API. The application is packaged as a single portable executable.

BeadWatch follows a three‐tier architecture: data acquisition (vendor database interface), processing and storage (metric computation and persistence) and presentation (interactive browser‐based dashboards) (Figure 1). The application is implemented in Python using FastAPI, with Chart.js for interactive data visualisation.

### 2.2 Data Storage

Application configuration (encrypted credentials, QC sample definitions, tracked bead selections and aliases) is stored in a local SQLite database co‐located with the executable, with JSON export/import for portability across instances.

Computed QC data (metrics, rolling statistics, alerts and caches) is stored in a dedicated SQL Server database on the same server as the vendor database. Co‐locating with the vendor instance simplifies deployment, as the existing SQL server infrastructure is reused and no additional server is required. The trade‐off is a dependency on the vendor server's availability, though this is acceptable given that BeadWatch already requires connectivity to that server for polling. BeadWatch's database is created and maintained automatically through idempotent migration scripts, and all access to the vendor database remains read‐only.

### 2.3 Vendor Adaptor Pattern

BeadWatch implements a pluggable vendor adaptor architecture. An abstract base class defines the required interface; concrete adaptors map vendor‐specific schemas to BeadWatch's normalised data model. The current release includes an adaptor for OneLambda HLA Fusion, the data management platform for OneLambda LABScreen bead‐based HLA antibody assays run on Luminex instruments. Adding vendor support requires implementing only the adaptor interface without modifying the core application.

### 2.4 Data Acquisition

A background scheduler polls the vendor database at a configurable interval (default: 60 min). New records are deduplicated, metrics are calculated (Section 2.5), checked against thresholds (Section 2.6) and rolling statistics and dashboard caches are updated incrementally.

### 2.5 QC Metrics

For each processed run, BeadWatch derives QC metrics from raw per‐bead and per‐well data. Some metrics are stored directly in the core per‐record metric table, while others are calculated in dedicated dashboard caches for QC sample, instrument and operator views.

#### 2.5.1 Control Metrics

- Positive control MFI (PC): the positive control bead MFI value per well.
- Negative control MFI (NC): the negative control bead MFI per well.
- Signal‐to‐noise ratio (S/N): PC/NC ratio for each well where NC > 0.

For trend display, per‐run control values are reported as the median across all wells, chosen over the mean for robustness to outlier wells (e.g., bubbles, incomplete aspiration).

#### 2.5.2 Bead Count Metrics

- Mean bead count: arithmetic mean of all per‐bead count readings in the core per‐record metric set.
- Median bead count: used in dashboard trend and cache views as a robust per‐run summary.
- Minimum bead count: lowest individual bead count.
- Low‐count percentage: percentage of beads with counts below 25, below which MFI measurements become unreliable [1].

#### 2.5.3 MFI Distribution Metrics

- Mean, median, standard deviation (n−1), min and max MFI across all per‐bead values.

#### 2.5.4 QC Sample Metrics

For runs containing user‐defined QC samples (identified by sample name or patient ID pattern matching):

- Median MFI across all beads.
- Median bead count.
- Per‐bead MFI stored as a JSON object mapping bead identifiers to individual MFI values, enabling longitudinal tracking of specific beads

### 2.6 Statistical Methods

#### 2.6.1 Rolling Statistics

For each metric, pre‐computed rolling statistics are maintained across four time windows: 7 days, 1 month, 1 year and all‐time. Statistics include mean, sample standard deviation (*n*−1), minimum, maximum and record count, updated incrementally after each polling cycle.

#### 2.6.2 LJ Chart Overlay

All trend charts display LJ reference lines computed from the selected time window:

- Mean line: arithmetic mean of per‐run median values.
- ±2SD lines: mean ±2 × sample standard deviation

Points exceeding ±2SD are highlighted. The Y‐axis is set to mean±3SD to maintain consistent scale while keeping outliers visible. Bessel‐corrected standard deviation (*n*−1) is used throughout, as each dataset represents a sample from an ongoing process.

#### 2.6.3 Individuals‐Moving Range (I‐MR) Charts

The Controls, Bead Counts and S/N Ratio charts offer an optional I‐MR sub‐chart. The moving range is the absolute difference between consecutive per‐run median values:

$$MR_{i}=\left|v_{i}-v_{i-1}\right|\;\text{for}\;i=1,\ldots ,n-1$$

Reference lines show the mean moving range ($\bar{\mathrm{MR}}$) and upper control limit (*UCL =* $\bar{\mathrm{MR}}$ *× D*_*4*_, where *D*_*4*_ *= 3.267* for subgroup size *n = 2*). This complements the LJ display: while the LJ chart flags values outside the expected range, the MR chart flags unusual run‐to‐run changes. For example, a step change after maintenance may remain within ±2SD but produce an MR spike exceeding the UCL.

#### 2.6.4 Outlier Detection

Two tiers of outlier detection operate concurrently:

Threshold‐based alerts. Configurable thresholds at warning and critical severity are checked against each new metric value for minimum bead count, mean MFI, signal‐to‐noise ratio and negative control MFI. Defaults were established from published recommendations and clinical experience. These settings can be adjusted per site.

QC sample outlier flagging. For QC samples grouped by catalogue, bead lot and role, mean and SD of median MFI are calculated across all runs. Results exceeding mean±2SD are flagged as outliers. This requires a minimum of five data points and non‐zero SD.

#### 2.6.5 Statistical Considerations

The ±2SD approach corresponds to the LJ 1_2s_ rule [10, 13], with an expected false positive rate of approximately 4.6% under normality. This is acceptable for a screening tool where flags prompt human review rather than automatic rejection. Several limitations apply:

- Multiplicity. Monitoring multiple metrics across instruments increases the family‐wise false alert rate. Multiplicity corrections (e.g., Bonferroni) were not applied as they would reduce sensitivity to genuine quality events.
- Self‐referencing. Reference lines are computed from the dataset being evaluated. For short windows (7 days) with few points, a single outlier can inflate the SD and mask its own detection.
- Minimum sample size. QC sample outlier detection requires a minimum of five data points; at this size, ±2SD boundaries are approximate screening limits.
- Normality assumption. S/N ratio may be right‐skewed as a ratio of two variables. No distributional transformation is applied (Section 4).

The Westgard multi‐rule system (1_3s_, 2_2s_, R_4s_, 4_1s_, 10 $\bar{\;x}$) [11, 13] is not implemented; it requires consecutive run tracking per instrument and metric, which adds non‐trivial architectural complexity.

### 2.7 Dashboard Features

The main dashboard provides four views (Controls, Bead Counts, S/N Ratio and QC Samples), each with selectable time windows (7 days, 1 month, 1 year, all‐time) and LJ overlays. Separate comparison dashboards plot S/N ratio, NC background and median bead count by instrument and operator against pooled reference lines (see Section 4.2 for rationale). The comparison dashboards use different windows suited to the analysis: 3 month, 1, 2, 3 year and all‐time. Active alerts display the metric, threshold breached, actual value and severity. Trend charts plot data points in run order on a category axis, with equal horizontal spacing per run regardless of elapsed time; visual compression or expansion within a time range therefore reflects variation in run frequency rather than the passage of time.

### 2.8 Deployment

BeadWatch is distributed as a portable Windows executable requiring no installation or administrator privileges. Microsoft ODBC Driver 18 for SQL Server is required and is provided separately as a prerequisite installer. A setup wizard guides initial configuration on first launch.

### 2.9 Testing

The test suite includes automated tests covering API endpoints, metric calculations, polling logic, threshold checking and caching. All tests are fully mocked, requiring no SQL Server connection.

### 2.10 Ethical Considerations

BeadWatch monitors instrument QC data using laboratory‐assigned sample identifiers rather than patient identifiers. Vendor database practices vary between sites, and some may store patient‐related identifiers in source systems; BeadWatch does not require these for its functionality. The QC sample identifiers used in BeadWatch are laboratory‐assigned codes, not NHS/patient numbers. Ethical approval was not required for the single‐site implementation described here.

## 3 Results

### 3.1 Feature Overview

Table 1 summarises BeadWatch's capabilities compared to common QC approaches.

**TABLE 1:** BeadWatch's capabilities compared with common QC approaches.

Feature	Manual spreadsheet	Vendor software (HLA fusion)	BeadWatch
Real‐time automated polling	No	No	Yes (configurable 5–1440 min)
Levey‐Jennings trend charts	Manual	No	Automated with ±2SD overlay
I‐MR (moving range) charts	No	No	Optional sub‐chart with UCL and auto‐interpretation
Bead count monitoring	Manual	Per‐run only	Longitudinal with low‐count alerts
S/N ratio trending	Manual	Per‐run display	Automated trending with outlier flagging
Per‐bead MFI tracking	No	No	Yes (user‐configurable tracked beads)
Cross‐instrument comparison	Manual	No	Automated scatter plot dashboard
Cross‐operator comparison	No	No	Automated scatter plot dashboard
QC sample longitudinal tracking	Manual	No	Automated with outlier detection
Configurable alert thresholds	N/A	N/A	Warning and critical levels per metric
Exportable charts	Manual	Limited	One‐click. PNG image file export
Installation required	N/A	Yes (full vendor suite)	No (single portable.exe)
Open source	N/A	No	Yes

### 3.2 Deployment Experience

We set up BeadWatch in our laboratory, which runs OneLambda LABScreen assays on FlexMap 3D Luminex instruments and previously operated IS100 instruments until 2024; results are imported into HLA Fusion for analysis. On first connection, approximately 20 years of historical QC data were immediately available; selected patterns identified from this data are described in (Section 3.3). The system has since run prospectively for 3 months at the time of writing.

**FIGURE 2:**
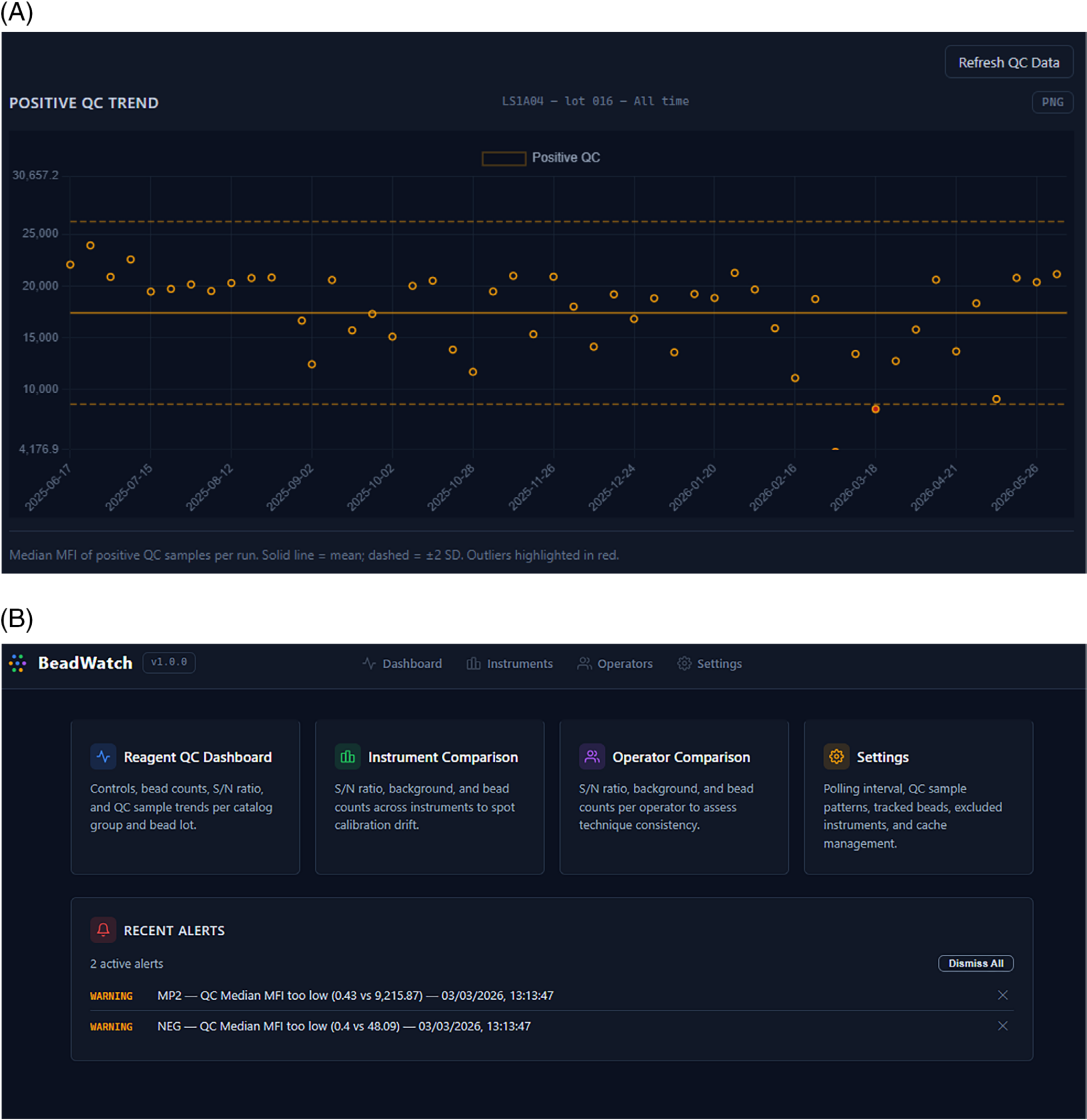
(A) Levey‐Jennings trend chart for the positive QC sample (MP2) showing near‐zero MFI on 3 March 2026, flagged as a warning by BeadWatch. Each circle is the per‐run median MFI. The solid horizontal line is the historical mean and the dashed lines indicate the ±2SD reference interval calculated from the same time window. (B) BeadWatch alerts panel on 3 March 2026 showing two simultaneous warnings: Near‐zero MFI for the positive QC sample (MP2: 0.43 vs. historical mean 9,215.87) and negative QC sample (NEG: 0.4 vs. 48.09). MP2 and NEG are local laboratory labels for the positive and negative QC samples respectively.

On 3 March 2026, shortly after an operator imported a run into HLA Fusion, BeadWatch raised alerts for near‐zero MFI for both the positive control (MP2: 0.43 vs. a historical mean of 9,215.87; Figure 2A,B) and negative control (NEG: 0.4 vs. 48.09; Figure 2B). The laboratory manager reviewed the alerts and confirmed with the operator that PE‐conjugate had been omitted from those sample wells. The operator had not yet identified the issue independently at the time the alerts appeared.

### 3.3 Observed QC Patterns

The following examples show QC patterns identified through BeadWatch during deployment. Across the monitoring period, we compared QC sample MFI between consecutive bead lots and observed no baseline shifts outside the ±2SD reference bands. Within individual lots, we also saw no progressive drift in positive or negative QC MFI that would indicate reagent degradation.

#### 3.3.1 Instrument Lifecycle Monitoring

**FIGURE 3:**
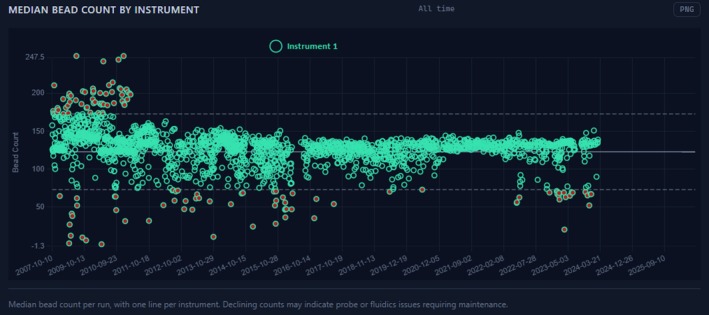
Bead count trend for a single IS100 instrument (anonymised as Instrument 1) over an 18‐year period (2007–2024). The solid line is the all‐time mean bead count and the dashed lines indicate the ±2SD reference interval calculated from the same window. Filled red circles mark points outside ±2SD. Three lifecycle phases are visible: Wide scatter with frequent high and low outliers in the early years (2007–2013); progressive stabilisation around the historical mean from approximately 2013 (2013–2023); and an increase in the frequency and severity of low‐count outliers from 2023 until decommissioning in 2024, following manufacturer end‐of‐life designation.

The bead count trend for a single IS100 instrument over an 18‐year period shows three distinct phases rather than isolated threshold events (Figure 3; x‐axis: category scale, spacing reflects run frequency). In the early years (2007–2013), bead counts showed wide scatter with frequent outliers both above and below ±2SD, including counts exceeding 200. From approximately 2013, variance progressively tightened and counts consolidated around the historical mean, remaining stable for the following decade. From 2023 onward, low‐count outliers became more frequent and more severe. The instrument was subsequently decommissioned in 2024 following manufacturer end‐of‐life designation.

#### 3.3.2 Operator Variability

**FIGURE 4:**
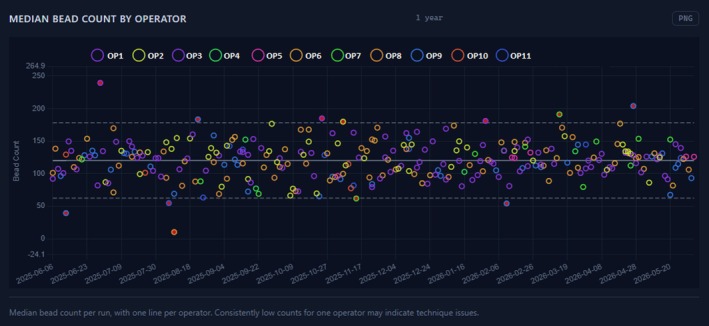
Operator comparison dashboard showing per‐run median bead counts for *N* = 11 operators, whose identifiers are anonymised to OP1–OP11, across the deployment period. The solid line is the pooled mean across all operators, and the dashed lines indicate the corresponding ±2SD reference interval. Outliers above and below ±2SD are scattered without a systematic per‐operator pattern, consistent with uniform technique across the team.

Figure 4 shows the operator comparison dashboard displaying bead counts per operator across the deployment period. No systematic between‐operator differences are apparent: Counts are distributed consistently across the team, with individual outliers scattered without pattern across operators. This finding reflects consistent operator technique and adherence to protocol.

#### 3.3.3 Instrument Platform Transition

**FIGURE 5:**
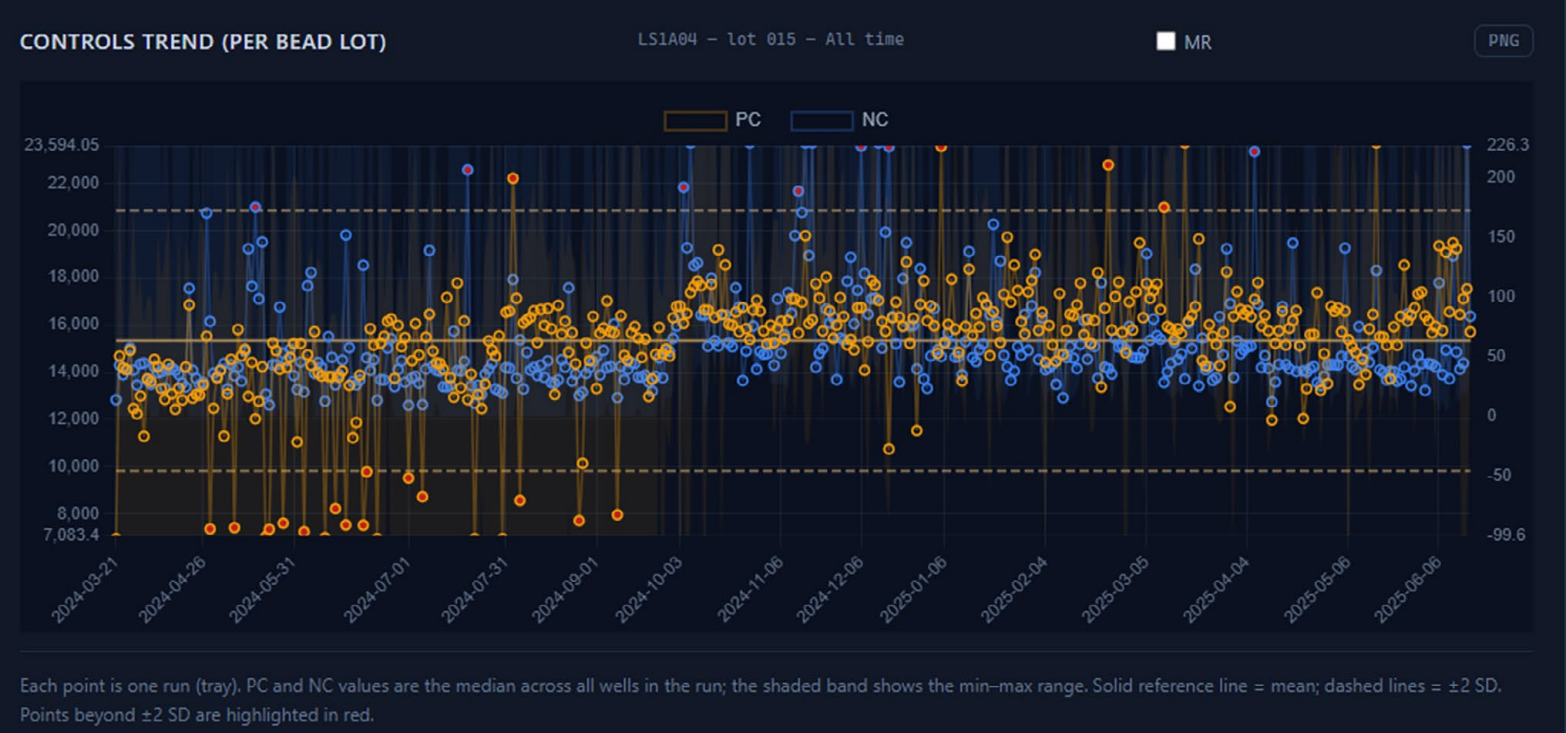
PC and NC controls trend spanning the laboratory's transition from IS100 to FlexMap 3D instruments in October 2024, with all instruments running the same bead lot (LS1A04 Lot 015) throughout. A step change in both PC and NC MFI is visible at the transition point, with higher values and tighter run‐to‐run variance on the FlexMap 3D platform. Outliers shown as filled red circles.

Figure 5 shows the PC and NC controls trend spanning the laboratory's transition from IS100 instruments to FlexMap 3D instruments in October 2024, with all instruments running the same bead lot throughout. A clear step change is visible at the transition point: post‐switch values for both PC and NC are systematically higher, while run‐to‐run variance is noticeably tighter.

The higher MFI on the FlexMap 3D platform is a known characteristic of the instrument's optical system; reagent vendors apply platform‐specific normalisation factors to account for this. BeadWatch captured the transition without any manual comparison, providing a quantitative record of the performance difference between platforms on identical reagents.

## 4 Discussion

### 4.1 Addressing the QC Gap

BeadWatch addresses a practical gap in QC infrastructure for H&I laboratories. Manual approaches (spreadsheet tracking and periodic visual review) can detect gross failures but are poorly suited to identifying gradual drift, between‐instrument divergence, operator variations and subtle reagent degradation that may affect clinical decisions before threshold violations occur.

The examples in Section 3 support this. The instrument lifecycle example (Section 3.3.1) illustrates how longitudinal monitoring surfaces multi‐phase patterns: early instability, a decade of stable operation, and a late‐career increase in low‐count outliers, which are invisible in per‐run review. During prospective use, at least one QC event was flagged before the responsible operator had independently identified the issue, suggesting automated monitoring can complement manual practices.

Because BeadWatch reads directly from the vendor database, historical data is immediately available without an accumulation period. At our site, this provided approximately 20 years of retrospective QC data on deployment.

### 4.2 Design Decisions

Median over mean for per‐run aggregation: Individual wells can exhibit outlier values from bubbles, incomplete aspiration or probe positioning. The median reflects typical performance; min‐max range bands provide visibility into the full spread.

Sample standard deviation (n–1): Bessel‐corrected SD is used throughout, appropriate when the dataset is a sample from an ongoing process.

±2SD rather than ±3SD: The ±2SD threshold was chosen as a screening boundary consistent with the LJ 1_2s_ rule, more sensitive than ±3SD and appropriate where flagged values are reviewed by staff rather than triggering automatic rejection.

Pooled cross‐instrument reference lines: The instrument comparison dashboard computes statistics from all instruments combined, revealing between‐instrument differences as displacements from the shared reference. Per‐instrument statistics would centre each on its own mean, obscuring the comparisons the dashboard is designed to surface.

Patient‐independent monitoring: ISO 15189 permits trend analysis of patient results as an alternative where appropriate IQC material is unavailable, but in H&I testing this is confounded by the laboratory's fluctuating clinical case mix (e.g., a batch of tests for highly sensitised patients would artificially spike a moving average). BeadWatch monitors only internal variables (PC, NC, S/N ratio, bead counts), isolating instrument and reagent performance from patient‐derived variation.

LIMS‐agnostic deployment: BeadWatch does not integrate directly with laboratory information management systems (LIMS). Coupling to a specific LIMS would tie deployment to that vendor and exclude laboratories using a different LIMS or none at all. QC trends can be exported as PNG for inclusion in any QC record or report system, preserving compatibility with any local workflow.

### 4.3 Regulatory Alignment

BeadWatch's features align with the accreditation requirements discussed in (Section 1.1): LJ trend analysis with rolling statistics across multiple time windows, I‐MR charts for run‐to‐run variability, automatic control extraction, configurable alerting and cross‐instrument/operator comparison dashboards. The system does not replace a laboratory's QC policy but may help evidence existing practices with reduced manual effort. Validation against specific accreditation requirements would need to be performed by each adopting laboratory.

### 4.4 Statistical Trade‐Offs

As a screening tool that prompts human review rather than triggering automatic rejection, BeadWatch accepts several statistical trade‐offs. These are detailed in Section 2.6.5; the key implications are that the single‐rule (1_2s_) approach is less discriminating than a full Westgard multi‐rule system, multiplicity across metrics and instruments inflates the false alert rate and self‐referencing reference lines can mask outliers in short time windows with few data points. The S/N ratio may also violate the normality assumption underlying ±2SD boundaries.

### 4.5 Other Limitations

Several limitations should be acknowledged. BeadWatch has been deployed and validated at a single laboratory, so its portability across other Fusion versions and operator workflows has not yet been independently demonstrated. Vendor support is currently limited to OneLambda HLA Fusion, although the pluggable adaptor architecture (Section 2.3) provides a defined extension path for additional platforms such as MatchIt! Antibody (Werfen); the effort required for any specific platform will depend on the structure of its underlying database.

BeadWatch is currently distributed for Windows only; versions for Mac or Linux have not been packaged.

The security model encrypts credentials locally, but does not implement user authentication, role‐based access or audit logging, so credential storage and network access to clinical databases warrant local risk assessment.

### 4.6 Future Directions

Several directions remain open. Multi‐site validation against different Fusion database versions and operator workflows would be the most informative next test, both to establish broader generalisability and to surface site‐specific configuration needs. The pluggable adaptor architecture supports additional vendor backends, for example, MatchIt! Antibody (Werfen), where the database schema permits. The same QC metrics could in principle apply to PCR‐SSOP HLA typing on the Luminex xMAP platform. Statistical extensions such as multi‐rule SPC approaches [11, 13] are natural directions for further work. So are optional per‐LIMS integration plugins that preserve the LIMS‐agnostic core design discussed in Section 4.2. Because BeadWatch is openly available, any of these extensions can be pursued, adapted or validated by users to suit local requirements.

### 4.7 Availability

BeadWatch is freely available at https://github.com/oxfordgenes/BeadWatch under the MIT Licence with Commons Clause. The repository includes source code, tests, executable releases, build instructions and documentation. Contributions and bug reports are welcomed via the Github issue tracker.

## 5 Conclusions

BeadWatch fills a practical gap in QC infrastructure for H&I laboratories, where accreditation requirements for statistical trend analysis often exceed what vendor software and manual approaches can deliver. Single‐site deployment demonstrated that automated trend monitoring can surface clinically relevant QC events before they are detected through routine review, with retrospective analysis extending visibility to historical data accumulated prior to deployment.

Multi‐site validation is needed to establish broader generalisability. The system is openly available to support adoption, community contributions and further evaluation.

## Author Contributions

M.C. and M.C.N.B. conceptualised the study and designed the system. M.C. developed the software, performed the analysis and drafted the manuscript. H.D., D.W., G.C. and M.Q.C. contributed to software testing, provided feedback on system functionality and assisted with manuscript review and editing. All authors approved the final version of the manuscript.

## Conflicts of Interest

The authors declare no conflicts of interest.

## Data Availability

The software described in this study is openly available at https://github.com/oxfordgenes/BeadWatch. The datasets generated and/or analysed during the current study are not publicly available due to institutional data governance restrictions.
